# Epigenetic Regulation of Regulated Cell Death in Aging-Related Diseases: Clinical Perspectives

**DOI:** 10.14336/AD.2025.1098

**Published:** 2026-10-22

**Authors:** Le Liu, Chen Li, Youshuo Liu

**Affiliations:** ^1^Department of Geriatrics, The Second Xiangya Hospital, Central South University, Changsha, Hunan, China.; ^2^Institute of Aging and Age-related Disease Research, Central South University, Changsha, Hunan, China.

**Keywords:** regulated cell death, epigenetic modification, aging, aging-related disease

## Abstract

The intricate crosstalk between epigenetic modification and regulated cell death (RCD) constitutes a pivotal yet underexplored axis in aging and its associated diseases. This Perspective conceptualizes age-related epigenetic reprogramming as a master switch that recalibrates the execution thresholds of diverse RCD pathways—including pyroptosis, ferroptosis, cuproptosis, necroptosis, and autophagy-dependent cell death—across multiple organ systems. We systematically decode how this epigenetic-RCD axis drives the pathophysiology of major aging-related conditions, such as diabetes, neurodegenerative disorders, cardiovascular diseases, and cancer, by synthesizing evidence of how DNA methylation, histone modifications, chromatin remodeling, non-coding RNAs, and RNA methylation intricately govern RCD networks. Building upon this mechanistic framework, the therapeutic potential of targeting this axis is critically examined, highlighting both emerging opportunities and translational challenges for future intervention strategies. Our analysis provides a novel paradigm for understanding aging mechanisms and proposes a roadmap for developing next-generation therapeutics.

## 1. Introduction

Aging entails a progressive deterioration of bodily functions that predisposes individuals to numerous aging-related diseases. With advancing age, the risk of developing conditions such as cancer, sarcopenia, neurodegenerative diseases, cardiovascular and metabolic disorders, as well as overall mortality, significantly increases [1-3]. The mechanisms underlying aging are multifaceted and include genomic instability, cellular senescence, telomere attrition, epigenetic alterations, loss of proteostasis, disabled macroautophagy, stem cell exhaustion, deregulated nutrient sensing, altered intercellular communication, mitochondrial dysfunction, chronic inflammation, and dysbiosis [4, 5]. These interconnected processes contribute to the progressive deterioration of cellular and tissue homeostasis, ultimately driving the aging phenotype and the onset of aging-related pathologies [6]. Elucidating these mechanisms in greater detail paves the way for the development of effective therapeutics aimed at mitigating aging and its accompanying diseases.

Regulated cell death (RCD) is an orchestrated form of cellular demise, governed by genetic programs that are crucial for maintaining internal homeostasis [7, 8]. Beyond classical apoptosis, major non-apoptotic RCD modalities, including pyroptosis, ferroptosis, cuproptosis, autophagy-dependent death, and necroptosis, exhibit distinct molecular signatures and functional outcomes [9, 10]. While these pathways collectively maintain tissue homeostasis, their age-related dysregulation drives functional decline and disease progression [11]. Notably, the epigenetic regulation of RCD may provide novel therapeutic opportunities for aging intervention.

Epigenetic regulation involves heritable modifications that modulate gene expression without changing the DNA sequence. It acts as a key interface between genetic programs and environmental factors. Major epigenetic mechanisms include: (1) DNA methylation [12, 13], (2) histone modifications [14], (3) chromatin remodeling [15], (4) non-coding RNAs [16], and (5) RNA modification [17], such as N6-methyladenosine (m6A) RNA methylation. Aging-related dysregulation of epigenetic modifications specifically controls RCD pathways, constituting a critical mechanism underlying aging and associated pathologies (Fig. 1) [7, 18, 19]. This Perspective charts the landscape of a central, actionable paradigm in aging biology: the ‘epigenetic-RCD axis’. We will first delineate the core molecular mechanisms that constitute this axis, then trace its disruptive influence across a spectrum of aging-related diseases to reveal a common, targetable driver of pathology. Finally, we evaluate emerging therapeutic strategies, highlighting the critical need for tissue-specific interventions to transform RCD from a passive endpoint in cell fate into a regulable node in disease progression, thereby paving the way for novel approaches to aging intervention and disease therapy.

**Figure 1.**
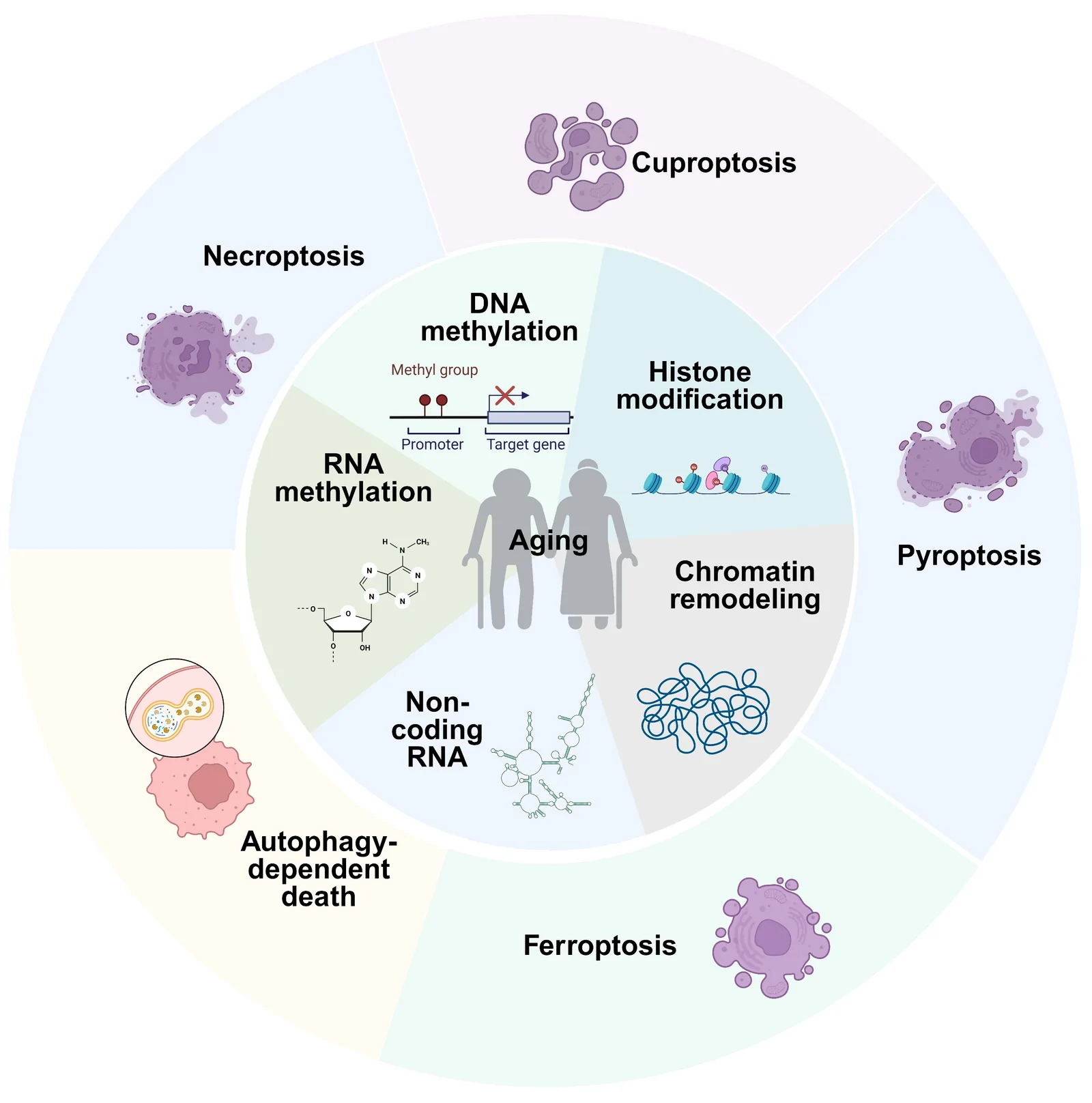
**Epigenetic regulation of RCD during aging**. During the aging process, alterations in epigenetic modifications, encompassing DNA methylation, histone modifications, chromatin remodeling, non-coding RNAs, and RNA modifications, regulate the advancement of RCD pathways. These pathways include pyroptosis, ferroptosis, cuproptosis, autophagy-associated cell death, and necroptosis. The mechanisms depicted are supported by evidence from a range of cellular and animal models of aging, utilizing techniques such as ChIP-seq, MeRIP-seq, and genetic/epigenetic editing.

## 2. Molecular mechanisms of RCD

Pyroptosis, ferroptosis, and other forms of regulated cell death (RCD) are characterized by distinct effector molecules and signaling pathways (Table 1). This section will systematically elucidate their regulatory mechanisms, thereby establishing the foundation for subsequent epigenetic regulation analysis in aging contexts.

### 2.1 Pyroptosis

Pyroptosis is a programmed cell death process initiated by inflammasomes and mediated by gasdermin proteins, which ultimately leads to cell lysis through pore formation in the plasma membrane [20]. Its hallmark features include cellular swelling, subsequent membrane disintegration, and the release of pro-inflammatory cytokines along with intracellular contents [21, 22].

**Table 1:** Comparison between pyroptosis, ferroptosis, cuproptosis, autophagy-dependent death, and necroptosis.

Type	Pyroptosis	Ferroptosis	Cuproptosis	Autophagy-dependent death	Necroptosis
**Inducing factors**	PAMPs/ DAMPs, Microbial infection	High extracellular iron, GPX4 inhibition, and ROS generation, Glutathione depletion	excessive intracellular copper accumulation	Oxidative stress, amino acid starvation, rapid declines in trophic factors or hormones, lipid starvation.	TNF-α, FasL, TRAIL, and viral infection
**Key molecules**	Caspase-1/4/5/11, GSDMD, NLRP3, ASC, IL-1β, IL-18	GPX4, SLC7A11, ACSL4, TFR1.	FDX1, DLAT, DLST, LIAS, SLC31A1	ATG, Beclin-1, LC3, ULK1, mTOR	RIPK1, RIPK3, MLKL
**Cellular morphology**	Cells swelling, pore formation, rupture and bubbling of plasma membranes.	Cells swelling, pore formation, decreased mitochondria crista, and rupture of mitochondrial outer membrane	Cell swelling, loss of mitochondrial cristae and rupture of the outer mitochondrial membrane	Accumulation of numerous autophagic vacuoles / autophagosomes, cytoplasmic vacuolization	Cell swelling, pore formation, rupture of plasma membranes, cytoplasmic and organelle swelling
**Biochemical events**	Assembly of inflammasome, caspase-mediated GSDM cleavage, mature IL-1β/IL-18 releases.	Iron accumulation, accumulation of lipid peroxides, membrane lipid peroxidation, GPX4 inactivation	Intracellular Cu accumulation, aggregation of DLAT, iron-sulfur cluster protein loss, proteotoxic stress	Activation of ULK1 complex, formation of autophagosomes, autophagosome-lysosome fusion	RIPK1/RIPK3 necrosome assembly; MLKL phosphorylation; membrane rupture.
**Release of cellular contents**	Cellular content, DAMPs (IL-18, IL-1β, HMGB1, ATP)	DAMPs (KRAS-G12D, HMGB1, 8-OHG, PGE2)	DAMPs (mtDNA, ATP, HMGB1, calreticulin)	Usually, no release	Cellular content, DAMPs (IL-1α, IL-33, IL-6, HMGB1, ATP, HSPs)

Pyroptosis is primarily orchestrated through four major signaling pathways: (1) The canonical inflammasome pathway: Activation of pattern recognition receptors leads to recruitment of pro-caspase-1, either directly by caspase activation and recruitment domain (*CARD*)-containing pattern recognition receptors or indirectly via adapter apoptosis-associated speck-like protein containing a *CARD* (ASC), forming caspase-1-dependent inflammasomes [20, 23, 24]. The assembled inflammasome then mediates the auto-cleavage and activation of caspase-1. Active caspase-1 subsequently cleaves two key substrates: the inactive precursors of IL-1β/IL-18, and GSDMD. Cleavage of GSDMD releases GSDMD-NT, which forms pores in the plasma membrane. These pores ultimately lead to pyroptosis and the release of inflammatory cytokines [25]. (2) The non-canonical pathway: Intracellular lipopolysaccharides (LPS) activate caspase-4/5 (caspase-11 in mice), cleaving GSDMD to induce K^+^ efflux, which triggers NLRP3 inflammasome assembly and pyroptosis [26]. Separately, caspase-11 cleaves pannexin-1 to release ATP, activating P2X7 receptor-mediated pyroptosis [27]. (3) Caspase-3/8-mediated pathway: Certain apoptotic caspases can trigger pyroptosis, with caspase-3 cleaving GSDME and caspase-8 cleaving both GSDMC and GSDMD to induce pyroptosis [28-30]. (4) Granzyme-mediated pathway: Upon delivery into target cells via perforin, granzymes can initiate pyroptosis by mediating the proteolytic cleavage of specific gasdermin family members [25]. This pathway is exemplified by granzyme A from cytotoxic T lymphocytes, which cleaves GSDMB to form membrane pores and induce pyroptosis in target cells expressing this gasdermin [31]. Similarly, chimeric antigen receptor T cells release granzyme B to trigger the caspase-3/GSDME pyroptosis pathway, resulting in extensive pyroptotic cell death [32].

### 2.2 Ferroptosis

Ferroptosis represents a distinctive form of RCD that is iron-dependent and features the toxic buildup of lipid peroxides, resulting in oxidative destruction of cellular membranes [33]. This process in mammalian cells is governed by three key biological processes: iron homeostasis, lipid metabolism, and redox balance [34].

Iron homeostasis: The pivotal role of iron overload in propelling ferroptosis is well established, with the processes of iron absorption, storage, and utilization constituting essential components of its regulation [34]. Excess iron accumulates in the labile iron pool. When iron metabolism is dysregulated, the labile iron pool expands, leading to increased production of reactive oxygen species (ROS) through Fenton reactions, which subsequently causes cellular damage or death [35]. In circulation, iron primarily exists in the ferric state (Fe³^+^) bound to transferrin [36]. Following its transferrin receptor 1 (TFR1)-mediated entry into cells, ferric iron is converted to its ferrous state (Fe²^+^) within endosomes through the action of STEAP3 [37]. The cytosolic release of endocytosed Fe²^+^, mediated by SLC11A2, serves as the source of the labile iron pool. This pool subsequently mediates hydroxyl radical formation, ultimately triggering ferroptosis [35].

Lipid metabolism: Polyunsaturated fatty acid chains (PUFAs) are highly susceptible to undergoing lipid peroxidation [38]. While free PUFAs do not directly drive ferroptosis, they must be esterified into membrane phospholipids to become lethal upon peroxidation [39, 40]. ACSL4 and lysophosphatidylcholine acyltransferase 3 are responsible for the biosynthesis and esterification of PUFA-PLs. These enzymes play a critical role in facilitating the esterification of PUFAs into PLs, thereby generating PL-PUFAs and promoting ferroptosis [34, 41]. The oxidation of membrane PUFAs into lipid peroxides (PUFA-OOH) by Fenton reaction-derived ROS instigates widespread cellular damage, including membrane destabilization and macromolecular injury, thereby executing ferroptosis [42].

Redox system homeostasis: Ferroptosis arises from the dysregulation of the delicate balance between the antioxidant defense system and intracellular oxidative stress [42]. Glutathione (GSH), the predominant cellular reductant, plays pivotal roles in Fe-S cluster assembly and serves as a central cofactor for various enzymes. Most critically, it enables GPX4 to reduce phospholipid hydroperoxides (PLOOHs) into non-toxic phospholipid alcohols (PLOHs), thereby preventing the oxidative membrane damage [43, 44]. Cellular cystine uptake occurs through the cystine/glutamate antiporter, followed by its reduction to cysteine via either GSH-dependent or thioredoxin reductase 1-mediated pathways to support GSH biosynthesis [43]. Disruption of the cystine/glutamate antiporter components impairs cystine uptake. This impairment subsequently limits GSH production and ultimately induces ferroptosis [45].

### 2.3 Cuproptosis

Cuproptosis is a recently discovered novel modality of cell death characterized by excessive intracellular copper (Cu) accumulation. This process is triggered when Cu induces the aggregation of mitochondrial enzyme dihydrolipoamide S-acetyltransferase (DLAT), disrupting the TCA cycle and Fe-S cluster proteins, ultimately leading to proteotoxic stress and cell death [46]. Cu is absorbed by intestinal epithelial cells through a protein called SLC31A1 [47]. Following intestinal absorption, copper enters the bloodstream and is systemically distributed by a dedicated transport complex. This complex, formed by ATP7A (ATPase Cu-transporting alpha) and its beta subunit, is central to this process, ensuring Cu delivery throughout the body [48]. Elevated intracellular copper prompts the translocation of ATP7A and ATP7B from the trans-Golgi network to intracellular vesicles. The fusion of these vesicles with the plasma membrane then facilitates the expulsion of Cu from the cell [49, 50]. Thereby, it effectively eliminates excess cytosolic Cu, safeguarding the cell from toxic accumulation. Cu^2+^ can be reduced to Cu^+^ by the mitochondrial matrix reductase ferredoxin 1 (FDX1) [51]. The aggregation of lipoylated DLAT, a central TCA cycle enzyme, is induced by direct binding with excess mitochondrial Cu^+^, which underlies the ensuing cytotoxicity [46]. In parallel, an excess of mitochondrial Cu^+^ compromises the integrity of Fe-S cluster proteins, an effect that also contributes to cell death [51]. Cu overload-induced cellular damage has also been associated with apoptosis. A primary mechanism of Cu ion cytotoxicity involves ROS generation through Fenton reactions. The resultant ROS overproduction mediates apoptotic cell death through several pathways, such as inducing mitochondrial damage, activating death receptors, and aggravating endoplasmic reticulum stress [48, 52].

### 2.4 Autophagy

Autophagy represents a process for the degradation of cellular constituents, playing an indispensable role in maintaining cellular homeostasis [53]. It is primarily categorized into three distinct forms: microautophagy, macroautophagy, and chaperone-mediated autophagy [54]. Macroautophagy (hereafter autophagy) entails the engulfment of cytoplasmic contents by specialized double-membrane vesicles named autophagosomes [55, 56]. These vesicles subsequently fuse with lysosomes, leading to the degradation of their contents into basic molecular components. More than 40 autophagy-related genes participate in autophagy [56]. Autophagy initiation is regulated by mechanistic target of rapamycin (mTOR) signaling pathways, which respond to cellular stress conditions [55]. The initiation of autophagy requires the dissociation of mTOR from ULK1. Following this, ULK1 is dephosphorylated at Ser757 by PP2A and PPM1D. Concurrently, AMPK mediates phosphorylation at Ser317 and Ser777, and these coordinated modifications promote ULK1 complex assembly [57-59]. The activated ULK1 complex induces PIKfyve phosphorylation at Ser1548, converting PI to PI (5) P. This conversion enables PI (5) P to interact with WIPI2, thereby promoting the separation of the isolation membrane from the ER membrane [60-62]. Membrane expansion requires the PI3KC3 complex to associate with both the ATG5-12 conjugate and ATG8/LC3 system through WIPI2, establishing a positive feedback loop that enhances their ubiquitination until complete autophagosome formation [63, 64]. Following deacetylation, STX17 anchors its C-terminal hairpin domain into mature autophagosomes. Once anchored, STX17 drives the binding between autophagosomes and lysosomes by interacting with SNAP29 and the HOPS complex. Lysosomal processing of the internalized cargo then follows, generating recyclable metabolites [65, 66].

### 2.5 Necroptosis

Necroptosis represents an RCD pathway mediated by the kinase activity of receptor-interacting serine/threonine-protein kinase 1 (RIPK1), which orchestrates the assembly of complex IIB to execute cell necroptosis [8]. When necroptosis occurs, active RIPK1 is recruited within an oligomeric complex comprising FADD, caspase-8 and caspase-10 [67]. When caspase-8 is deficient, RIPK1 serves as the critical trigger that recruits and phosphorylates RIPK3, culminating in ripoptosome formation [68]. The RIPK1/RIPK3 complex recruits and phosphorylates MLKL, a core event that initiates necrosome assembly [69]. MLKL oligomers subsequently relocate to plasma membrane regions enriched in phosphatidylinositol phosphate, where they assemble large membrane pores [68]. Subsequently, increased plasma membrane permeability results in membrane rupture and the release of damage-associated molecular patterns (DAMPs), culminating in necroptotic demise [70]. This cell death modality displays a unique morphological profile: perforated plasma membrane, osmotically driven cell rounding and swelling, enlarged organelles, mitochondria lacking membrane potential, diminished nuclear chromatin, and finally, explosive plasma membrane rupture [8].

## 3. Epigenetic mechanisms regulating RCD pathways in aging

Epigenetic mechanisms dynamically orchestrate gene expression patterns in the absence of alterations to the underlying DNA sequence, fundamentally directing cellular identity, differentiation, proliferation, survival, and death - thereby determining cell fate. These mechanisms, including DNA methylation, histone modifications, chromatin remodeling, non-coding RNAs, and RNA modifications, etc., function synergistically to establish and maintain specific transcriptional programs [71, 72].

Crucially, epigenetic regulation plays a vital role in regulated cell death (RCD) pathways. Specifically, the H3K4-specific histone methyltransferase WDR5 and DOT1L regulate *NLRP3* expression, thereby modulating pyroptosis [73]. Furthermore, METTL14 inhibits pyroptosis by increasing N6-methyladenosine (m6A) methylation of the *TINCR* transcript, thereby reducing its expression levels [74]. METTL3 mediates m6A methylation on the *SLC7A11* transcript, which is subsequently recognized by YTHDF2 to promote mRNA destabilization, thereby collectively regulating ferroptosis [75]. The H3K9 demethylase KDM3B promotes *SLC7A11* transcription by lowering H3K9me levels at its promoter. Conversely, BAP1 and PRC1 inhibit *SLC7A11* expression by deubiquitinating H2A at its promoter, consequently modulating ferroptosis [76]. METTL16 mediates cuproptosis by regulating m6A methylation of *FDX1* mRNA [77]. Necroptosis-related factors are tightly regulated epigenetically by SIRT3 and various non-coding RNAs [7]. Furthermore, the Sin3A complex containing HDAC1/2 suppresses autophagy-related gene expression by removing acetylation from histone H4 [78], while the m6A reader protein YTHDF3 promotes autophagy induction by upregulating *FOXO3* mRNA translation [79].

Aging is accompanied by epigenetic alterations. With advancing age, the expression level of *DNMT1* decreases, leading to reduced global DNA methylation [18]. During cellular senescence, chromatin structure undergoes significant remodeling, involving alterations ranging from changes in histone composition and modifications to reorganization of chromatin compartments and topologically associating domains [18, 80, 81]. Furthermore, m6A RNA methylation represents a key regulatory layer influencing the aging process [82, 83]. The interplay between epigenetic modifications and RCD critically modulates aging processes. Cu overload exacerbates testicular aging mediated by deficiency of the lncRNA CR43306 through ferroptosis [84]. Additionally, lncRNA UCA1 delays cardiomyocyte senescence via concurrent suppression of oxidative stress and ferroptosis [85]. Furthermore, the demethylase FTO induces m6A demethylation of autophagy-related genes (*ATG5/ATG7*). This demethylation leads to the YTHDF2-dependent upregulation of *ATG5 and ATG7* expression. The increased levels of these autophagy proteins enhance cellular autophagy and attenuate senescence [86]. Collectively, these findings establish the strategic reprogramming of the epigenetic landscape as a viable approach to recalibrate RCD pathways and mitigate tissue-specific aging.

## 4. Epigenetic modification of RCD in aging-related diseases

Epigenetic dysregulation leading to aberrant control of regulated cell death (RCD) pathways serves as a key driver of aging-related diseases. Age-associated epigenetic alterations profoundly impact the activation or suppression of key RCD pathways, including pyroptosis, ferroptosis, cuproptosis, autophagy, and necroptosis. This disruption at the epigenetic level, by altering the balance of RCD, plays a pivotal role in the pathogenesis of major aging-related diseases such as metabolic disorders, cardiovascular diseases, neurodegenerative diseases, and cancer (Table 2, Fig. 2 and Fig. 3).

**Figure 2.**
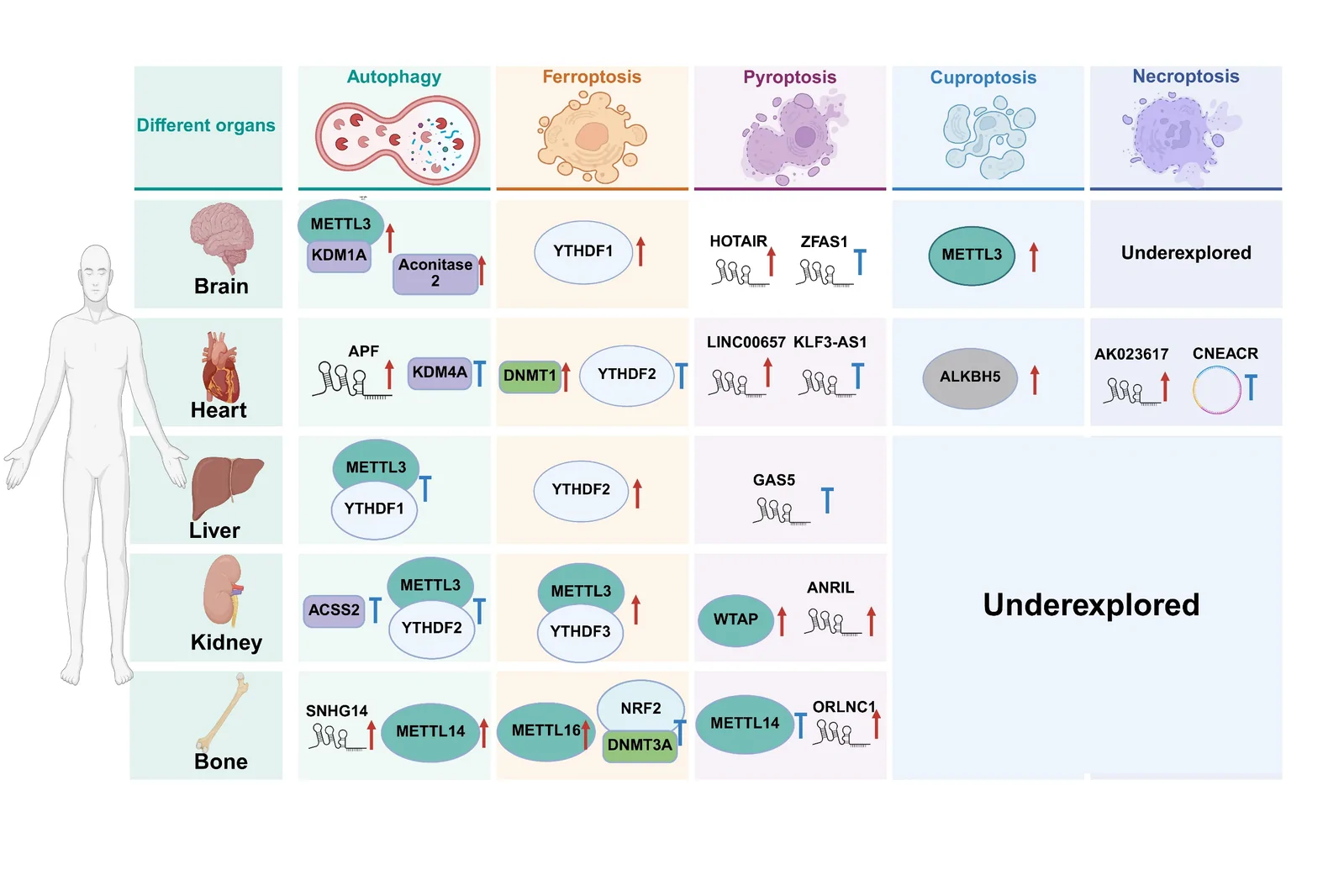
**Organ-specific epigenetic regulation of RCD in aging-related diseases**. During the aging process, epigenetic regulators such as METTL3, YTHDF1/2, KDM1A, DNMT1, and ANRIL modulate multiple RCD pathways, ultimately driving the progression of multi-system aging-related diseases in the brain, heart, liver, kidney, and bone tissue. All depicted regulatory effects of epigenetic regulators on RCD pathways represent experimentally validated causal relationships. The organ-specific findings summarized here are derived from studies employing relevant disease models, including those for Alzheimer's disease, Parkinson's disease, myocardial infarction, atherosclerosis, heart failure, myocardial fibrosis, NAFLD, diabetic kidney disease, and osteoporosis, utilizing techniques such as m6A-seq, luciferase reporter assays, and in vivo functional analysis.

### 4.1 m6A methylation of RCD in aging-related diseases

A central finding is the pivotal role of the N6-methyladenosine (m6A) methylation machinery in modulating aging-related diseases. The m6A methyltransferases such as METTL3, METTL14 and WTAP increase the m6A modification of key molecules in RCD pathways in an m6A reader-dependent manner [87-89] (e.g., YTHDF2, IGF2BPs), while the demethylases FTO and ALKBH5 play the opposite role [90, 91], thereby jointly regulating the occurrence of RCD, and ultimately affecting the pathogenesis and development of aging-related diseases.

m6A modification regulates autophagy in multiple diseases. For instance, in the liver, METTL3 binds to *RUBCN* mRNA and mediates its m6A modification. Simultaneously, the reader protein YTHDF1 recognizes this m6A mark and stabilizes the transcript by inhibiting its degradation. The resulting accumulation of RUBCN protein suppresses hepatic autophagy, which in turn promotes lipid accumulation [92]. Meanwhile, in cardiomyocytes, METTL3 suppresses protective autophagy by regulating *ATG7* transcript stability via an m6A-YTHDF2-dependent mechanism, exacerbating cellular injury [93]. METTL14 enhances *RB1CC1* expression via the m6A-IGF2BP2-dependent pathway, promoting autophagy and inhibiting tumor progression in oral squamous cell carcinoma [94]. In contrast, ALKBH5 promotes *VPS11* mRNA translation by removing m6A modifications, restoring autophagic flux and alleviating hepatic lipid deposition [95]. Meanwhile, ALKBH5 promotes senescent foam macrophage formation via the *CCL5/CCR5*/autophagy axis in atherosclerosis (AS) [96]. In myocardial fibrosis, the m6A reader protein YTHDF1 regulates cardiac fibroblast autophagy by recognizing the m6A modification on *PIEZO2* [97].

**Table 2:** Epigenetic Regulation of RCD in aging-related diseases.

RCD Pathway	Aging-related diseases type	Types of epigenetic modification	Major regulator	Effects on RCD
**Autophagy**	Diabetes mellitus	Non-coding RNA	MEG3	Induce autophagy
		RNA methylation	FTO	Induce autophagy
		DNA methylation	RCAN1	Inhibit autophagy
	Diabetic kidney disease	RNA methylation	METTL3/ YTHDF2	Inhibit autophagy
		Histone modification	ACSS2	Inhibit autophagy
		Non-coding RNA	SOX2OT	Induce autophagy
	Diabetic retinopathy	Non-coding RNA	FAT1	Induce autophagy
	Diabetic macrovascular complication	Non-coding RNA	LYPLAL1-DT	Induce autophagy
	Non-alcoholic fatty liver disease	RNA methylation	METTL3/ YTHDF1	Inhibit autophagy
		RNA methylation	ALKBH5	Induce autophagy
	Osteoporosis	Non-coding RNA	SNHG14	Induce autophagy
		RNA methylation	METTL14	Induce autophagy
	Atherosclerosis	RNA methylation	ALKBH5	Inhibit autophagy
		DNA methylation	DNMT1	Induce autophagy
		Non-coding RNA	lncARF	Inhibit autophagy
		Non-coding RNA	LINC01235	Inhibit autophagy
		Non-coding RNA	LUCAT1	Induce autophagy
		Non-coding RNA	RASSF8-AS1	Induce autophagy
		Non-coding RNA	MALAT1	Inhibit autophagy
		Non-coding RNA	THRIL	Induce autophagy
	Myocardial infarction	RNA methylation	METTL3	Inhibit autophagy
		Histone modification	KDM4A	Inhibit autophagy
		Non-coding RNA	MALAT1	Regulate autophagy bidirectionally
		Non-coding RNA	DANCR	Induce autophagy
		Non-coding RNA	PART1	Inhibit autophagy
		Non-coding RNA	APF	Induce autophagy
		Non-coding RNA	CAIF	Inhibit autophagy
		Non-coding RNA	TUG1	Induce autophagy
		Non-coding RNA	TTTY15	Inhibit autophagy
		Non-coding RNA	MIRF	Inhibit autophagy
	Heart failure	DNA methylation	DNMT1	Inhibit autophagy
		Non-coding RNA	MEG3	Induce autophagy
		Non-coding RNA	TUG1	Induce autophagy
	Myocardial fibrosis	RNA methylation	YTHDF1	Induce autophagy
		DNA methylation	DNMT3A	Induce autophagy
		Non-coding RNA	DCRF	Induce autophagy
	Alzheimer's disease	Histone modification	KDM1A	Induce autophagy
		RNA methylation	METTL3	Induce autophagy
		Non-coding RNA	EPB41L4A-AS1	Induce autophagy
	Parkinson's disease	Histone modification	Aconitase 2	Induce autophagy
		Non-coding RNA	OIP5-AS1	Induce autophagy
		Non-coding RNA	SNHG1	Inhibit autophagy
		Non-coding RNA	A2M-AS1	Induce autophagy
		Non-coding RNA	BDNF-AS	Induce autophagy
		Non-coding RNA	NEAT1	Induce autophagy
		Non-coding RNA	tRF-02514	Inhibit autophagy
	Oral squamous cell carcinoma	RNA methylation	METTL14	Induce autophagy
	Lung adenocarcinoma	Non-coding RNA	LOC85009	Inhibit autophagy
	Gastric cancer	Non-coding RNA	CRNDE	Inhibit autophagy
**Cuproptosis**	Myocardial infarction	RNA methylation	ALKBH5	Induce cuproptosis
	Alzheimer's disease	RNA methylation	METTL3	Induce cuproptosis
	Colorectal cancer	Non-coding RNA	PVT1	Induce cuproptosis
**Ferroptosis**	Diabetic kidney disease	RNA methylation	METTL3/ YTHDF3	Induce ferroptosis
	Diabetic retinopathy	RNA methylation	ALKBH5	Inhibit ferroptosis
	Diabetic cardiomyopathy	RNA methylation	ALKBH5	Induce ferroptosis
	Diabetic osteoporosis	DNA methylation	DNMT1/ DNMT3a	Induce ferroptosis
		RNA methylation	METTL3	Induce ferroptosis
	Diabetic liver injury	RNA methylation	YTHDF2	Induce ferroptosis
	Osteoporosis	RNA methylation	METTL16	Induce ferroptosis
		RNA methylation	ZC3H13	Inhibit ferroptosis
		DNA methylation	NRF2/DNMT3A	Inhibit ferroptosis
	Atherosclerosis	Non-coding RNA	NORAD	Inhibit ferroptosis
	Myocardial infarction	RNA methylation	ZC3H13	Inhibit ferroptosis
		RNA methylation	hnRNPA2B1	Induce ferroptosis
		RNA methylation	YTHDF2	Inhibit ferroptosis
		DNA methylation	DNMT1	Induce ferroptosis
		Histone modification	KMT2B	Induce ferroptosis
	Heart failure	Non-coding RNA	GAS5	Inhibit ferroptosis
	Parkinson's disease	RNA methylation	YTHDF1	Induce ferroptosis
		RNA methylation	FTO	Induce ferroptosis
	Sarcopenia	Non-coding RNA	GPRC5D-AS1	Inhibit ferroptosis
	Breast cancer	RNA methylation	METTL14	Induce ferroptosis
	Colorectal cancer	RNA methylation	FTO	Inhibit ferroptosis
	Gastric cancer	Non-coding RNA	CBSLR	Inhibit ferroptosis
	Non-small cell lung cancer	Non-coding RNA	RGMB-AS1	Induce ferroptosis
	Prostate cancer	DNA methylation	EZH2	Inhibit ferroptosis
		Histone modification	EZH2	Inhibit ferroptosis
**Necroptosis**	Atherosclerosis	Non-coding RNA	AK023617	Induce necroptosis
	Myocardial infarction	Non-coding RNA	circCacna1c	Inhibit necroptosis
		Non-coding RNA	CNEACR	Inhibit necroptosis
	Liver cancer	Non-coding RNA	HABON	Inhibit necroptosis
**Pyroptosis**	Diabetes mellitus	Non-coding RNA	MEG3	Inhibit pyroptosis
	Diabetic kidney disease	RNA methylation	WTAP	Induce pyroptosis
		Non-coding RNA	ANRIL	Induce pyroptosis
		Non-coding RNA	MALAT1	Induce pyroptosis
	Diabetic retinopathy	RNA methylation	RBM15	Induce pyroptosis
		RNA methylation	METTL3	Inhibit pyroptosis
		Non-coding RNA	FAT1	Inhibit pyroptosis
	Non-alcoholic fatty liver disease	Non-coding RNA	GAS5	Inhibit pyroptosis
	Osteoporosis	RNA methylation	METTL14	Inhibit pyroptosis
		Non-coding RNA	ORLNC1	Induce pyroptosis
	Atherosclerosis	RNA methylation	METTL3	Induce pyroptosis
		RNA methylation	WTAP	Induce pyroptosis
		Non-coding RNA	AU020206	Inhibit pyroptosis
		Non-coding RNA	linc00657	Induce pyroptosis
		Non-coding RNA	MALAT1	Induce pyroptosis
		Non-coding RNA	GAPLINC	Induce pyroptosis
		Non-coding RNA	AC078850.1	Induce pyroptosis
	Myocardial infarction	RNA methylation	ALKBH5	Induce pyroptosis
		RNA methylation	METTL14	Induce pyroptosis
		Non-coding RNA	TUG1	Induce pyroptosis
		Non-coding RNA	H19	Inhibit pyroptosis
		Non-coding RNA	KLF3-AS1	Inhibit pyroptosis
		Non-coding RNA	FAF	Inhibit pyroptosis
	Heart failure	RNA methylation	METTL14	Induce pyroptosis
		Non-coding RNA	HOTAIR	Inhibit pyroptosis
		Non-coding RNA	XIST	Inhibit pyroptosis
	Myocardial fibrosis	DNA methylation	DNMT3A	Induce pyroptosis
		DNA methylation	DNMT1	Induce pyroptosis
		Non-coding RNA	GAS5	Inhibit pyroptosis
	Parkinson's disease	Non-coding RNA	HOTAIR	Induce pyroptosis
		Non-coding RNA	ZFAS1	Inhibit pyroptosis
		Non-coding RNA	tRF-02514	Induce pyroptosis
	Breast cancer	Non-coding RNA	MEG3	Induce pyroptosis

The m6A epigenetic machinery is a key determinant of cellular fate towards ferroptosis in numerous aging-related diseases. For instance, METTL3 promotes ferroptosis by stabilizing *TFR1* in an m6A-YTHDF3-dependent manner in diabetic kidney disease (DKD) [98]; Meanwhile, under high-glucose/high-fat conditions, METTL3 activates osteoblast ferroptosis through the METTL3/ASK1-P38 signaling pathway [99]. Beyond METTL3, METTL16-mediated m6A modification of *PPARγ* mRNA induces ferroptosis in bone marrow mesenchymal stem cells (BMSCs), thereby inhibiting their osteogenic differentiation [100]. Notably, the m6A methyltransferase ZC3H13 positively regulates osteogenic capacity in osteoporotic BMSCs by modulating ferroptosis [101]. In contrast, FTO promotes neuronal ferroptosis by regulating both the NRF2 axis and the BAP1/p53/SLC7A11 axis through removal of m6A modifications [102, 103]. Meanwhile, ALKBH5-mediated demethylation of *SPOP* mRNA leads to suppressed VDAC3 ubiquitination, consequently promoting ferroptosis in diabetic cardiomyopathy (DCM) [104]. ZC3H13-mediated m6A modification inhibits inflammation and ferroptosis by targeting lncRNA93358, ameliorating MI [105]; while hnRNPA2B1 drives cardiomyocyte ferroptosis by recognizing the m6A modification site on *PFN2* mRNA and stabilizing it [106]. YTHDF2-mediated *NCOA4* m6A methylation regulates myocardial ferroptosis by suppressing *NCOA4* expression [107]. In the context of cancer, *METTL14* deficiency-induced m6A hypomethylation activates FGFR4, thereby alleviating ferroptosis through the β-catenin/TCF4-SLC7A11/FPN1 axis and driving HER2 resistance in breast cancer [108]; whereas FTO suppresses ferroptosis in colorectal cancer cells via the m6A-YTHDF2-dependent SLC7A11/GPX4 axis, consequently promoting tumorigenesis [109].

**Figure 3.**
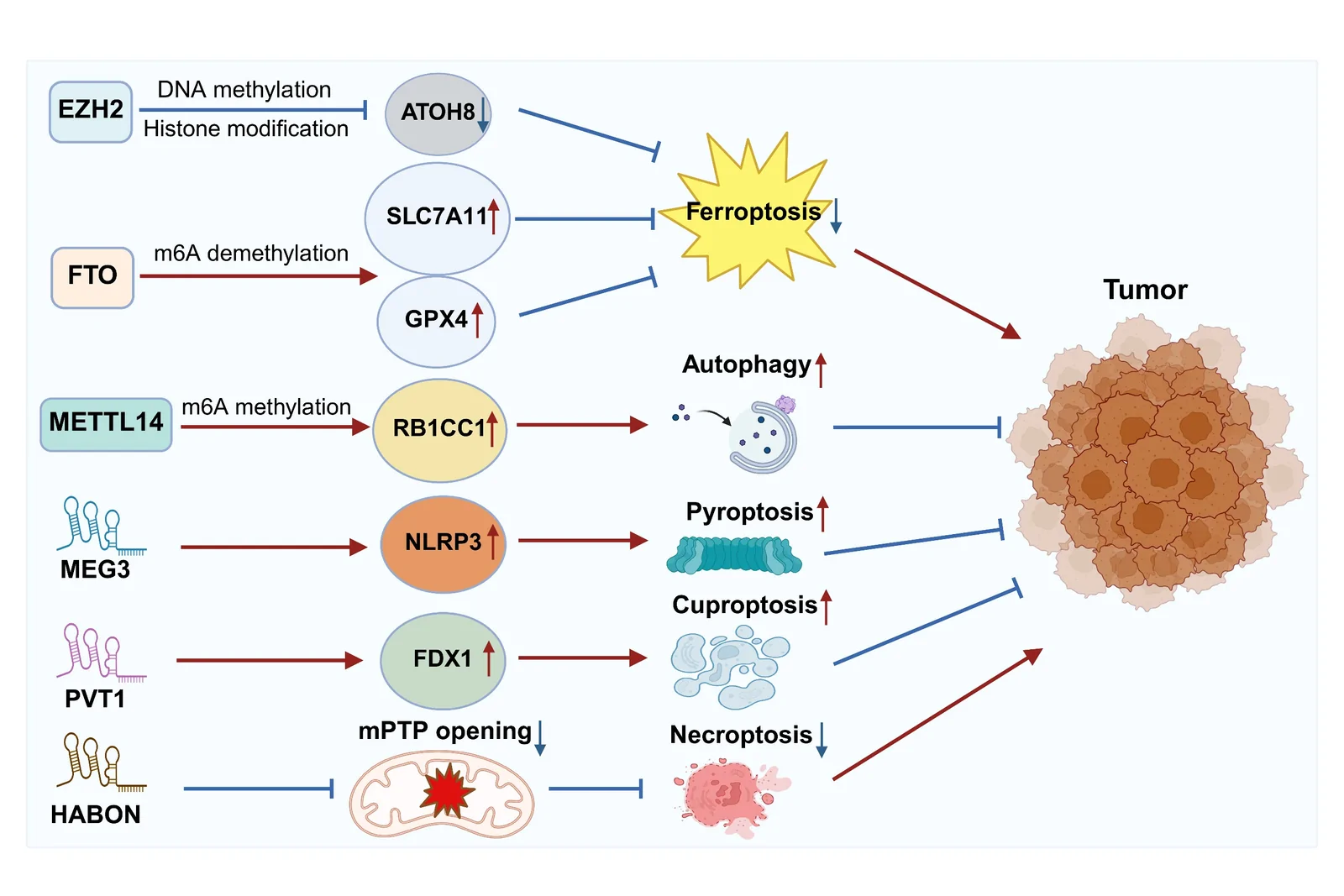
**Epigenetic regulation of RCD in cancer**. Epigenetic regulators including EZH2, FTO, METTL14, MEG3, PVT1 and HABON modulate pyroptosis, ferroptosis, cuproptosis, autophagy-dependent cell death, and necroptosis pathways, thereby influencing tumorigenesis. All regulatory axes shown are supported by causal evidence from experimental validation. Evidence is derived from studies across various cancer types, such as colorectal, gastric, and lung cancers, utilizing techniques including gain/loss-of-function experiments, RNA immunoprecipitation, and promoter methylation analysis.

The regulatory landscape of pyroptosis by m6A modification exhibits notable complexity, with different m6A modifiers exerting divergent effects depending on cellular and pathological context. For instance, METTL3 confers protection against high glucose-induced pyroptosis in retinal pigment epithelial cells by regulating the miR-25-3p/PTEN/AKT pathway via DGCR8 [110], whereas it exacerbates endothelial inflammation and pyroptosis in AS by enhancing m6A modification of lncRNA H19 [111]. METTL14-mediated m6A modification suppresses NLRP3-dependent macrophage pyroptosis and osteoclastogenesis in osteoporosis [112], yet it exacerbates cardiomyocyte pyroptosis through mechanisms involving pri-miR-5099 m6A methylation and the miR-221-3p/lncRNA FTX/SESN2 pathway [113, 114]. Similarly, WTAP promotes pyroptosis by upregulating *NLRP3* expression in atherosclerotic macrophages [115]. RBM15 stabilizes *KLF6* expression through enhanced m6A methylation of its mRNA, promoting pyroptosis and inflammatory responses in retinal ganglion cells [116]. Furthermore, ALKBH5 triggers cardiac fibroblast pyroptosis through the NOTCH1/NLRP3 axis [117] and, in a positive feedback loop with NLRP3, fosters cardiomyocyte pyroptosis [118].

### 4.2 Regulation of RCD by lncRNAs in aging-related diseases

A network of long non-coding RNAs (lncRNAs) provides precise, signal-integrated control in modulating aging-related diseases. They frequently function as epigenetic modifiers, establishing intricate regulatory axes that determine the thresholds of various RCD pathways. The ensuing context-specific dysregulation of these lncRNA-RCD networks drives the initiation and progression of pathologies across metabolic, cardiovascular, neurodegenerative, and oncological conditions.

lncRNAs have emerged as a potent driver in the regulation of autophagy. In metabolic disorders, MEG3 regulates autophagy in liver endothelial cells to maintain glucose homeostasis [119]. Separately, SOX2OT ameliorates the fibrotic phenotype in mesangial cells via the AKT/mTOR-mediated autophagy pathway [120]. Meanwhile, *SNHG14* activates autophagy in BMSCs through the miR-493-5p/MEF2C axis to retard osteoporosis [121]. In cardiovascular diseases, MALAT1 dynamically regulates cardiomyocyte autophagy in myocardial infarction (MI) [122-124]; and a spectrum of lncRNAs including LINC01235, LUCAT1, RASSF8-AS1, MALAT1, and THRIL modulate autophagic activity in various vascular cells to influence atherosclerotic progression [125-129]. Furthermore, MEG3 and TUG1 regulate cardiomyocyte autophagy in heart failure through distinct miRNA-129-5p-mediated pathways [130, 131], with additional lncRNAs such as DANCR, PART1, and CAIF contributing to autophagy regulation in myocardial injury and repair [132-138]. In Parkinson's disease (PD), OIP5-AS1, A2M-AS1 and downregulation of SNHG1 promote protective mitophagy [139-141], whereas BDNF-AS and NEAT1 drive autophagy-dependent apoptosis [142, 143]. The clinical relevance of lncRNA-mediated autophagy regulation is further evidenced in oncology, where LOC85009 suppresses docetaxel resistance in lung adenocarcinoma by modulating *ATG5*-dependent autophagy [144], and CRNDE regulates chemoresistance in gastric cancer through splicing regulation of *PICALM* mRNA [145]. These findings collectively highlight lncRNAs as critical context-dependent regulators of autophagic pathways in aging-related pathophysiology.

lncRNAs provide another crucial layer of regulation in ferroptosis across various aging-related pathologies. In AS, lncRNA NORAD promotes endothelial cell proliferation and suppresses ferroptosis via the miR-106a/CCND1 axis [146]. In heart failure, BMSC-derived exosomal lncRNA GAS5 suppresses cardiomyocyte ferroptosis by inhibiting the ULK1/HIPPO pathway [147]. In oncology, lncRNA-CBSLR regulates *CBS* expression via an m6A-YTHDF2-mediated regulatory pathway, influencing ferroptosis in gastric cancer cells[148]; while lncRNA RGMB-AS1 triggers ferroptosis in non-small cell lung cancer through the suppression of HMOX1 ubiquitination[149].

A sophisticated network of lncRNAs serves as precise regulators of pyroptotic signaling across aging-related diseases, demonstrating context-specific effects. In metabolic disorders, lncRNA ANRIL and MALAT1 drive pyroptosis in DKD through the miR-497/TXNIP and miR-30c/NLRP3 axes respectively [150, 151], while GAS5 suppresses hepatocyte pyroptosis via the miR-28a-5p/MARCH7/NLRP3 axis in non-alcoholic fatty liver disease (NAFLD) [114], and ORLNC1 promotes pyroptosis in osteoporotic BMSCs through the miR-200b-3p/FOXO3 axis [152]. In cardiovascular diseases, lncRNAs differentially regulate pyroptosis, with linc 00657, MALAT1, GAPLINC, AC078850.1, and TUG1 promoting its activation [153-157], while AU02 0206, H19, KLF3-AS1, FAF, HOTAIR, and XIST inhibit it [158-163]. Neurological and oncological contexts further illustrate this regulatory importance: in PD, HOTAIR promotes neuronal pyroptosis via the miR-326/ELAVL1 axis while ZFAS1 inhibits it through interfering with miR-590-3p [164, 165], and in triple-negative breast cancer, MEG3 induces pyroptosis via NLRP3/caspase-1/GSDMD activation [166]. These findings establish lncRNAs as master regulators of pyroptotic balance in aging-related pathophysiology.

### 4.3 Epigenetic modification of RCD in metabolic diseases

Metabolic diseases epitomize the systemic influence of the epigenetic RCD axis. In this condition, hyperglycemia, oxidative stress, and metabolic disturbances dynamically modulate key RCD molecules through epigenetic reprogramming, thereby orchestrating tissue-specific dysregulation of cell death pathways. This process underpins the pathogenesis of diverse diseases, including diabetes, NAFLD, and osteoporosis, ultimately driving multi-organ damage.

The dynamic balance of autophagy, crucial for cellular homeostasis, is extensively remodeled by the epigenetic landscape in metabolic diseases. Obesity-induced RCAN1 overexpression contributes to pancreatic β-cell failure by inhibiting mitophagy. This occurs through RCAN1-mediated hypermethylation of the *BECLIN*-1 gene, which in turn suppresses autophagy initiation. This ultimately leads to MIRO1-mediated mitophagy deficiency [167]. ACSS2 mediates histone H3K9 acetylation to epigenetically activate *RAPTOR* and enhance mTORC1 signaling, thereby suppressing protective autophagy and promoting podocyte injury [168]. Additionally, in vitro experiments demonstrate that circular RNA FAT1 modulates both pyroptosis and autophagy processes in retinal pigment epithelial cells by regulating the expression of *YTHDF2* [169].

Concurrently, epigenetically orchestrated ferroptosis emerges as a potent driver of tissue damage across metabolic tissues. In the liver, transcription factor ZHX2 suppresses ferroptosis through epigenetic silencing of *YTHDF2*—forming a ZHX2/YTHDF2 negative feedback loop—thereby alleviating diabetic liver injury. YTHDF2 recognizes m6A-modified *ZHX2* mRNA and reduces its stability; conversely, ZHX2 represses *YTHDF2* transcription by binding to its promoter region [170]. In addition, diabetic osteoporosis (DOP) model mouse femurs display ferroptotic features including suppressed *GPX4*, driven by DNMT1/DNMT3A-mediated *GPX4* promoter hypermethylation [171]. The transcription factor NRF2 regulates osteocyte ferroptosis and osteoclastogenesis through DNMT3A-mediated modulation of *RANKL* promoter DNA methylation levels, thereby influencing osteoporosis [172].

This collective evidence positions the epigenetic-RCD axis as a key driver of metabolic diseases. Targeting this maladaptive epigenetic reprogramming is thus critical to break the vicious cycle of metabolic memory and thereby alleviate tissue damage.

### 4.4 Epigenetic modification of RCD in cardiovascular diseases

The cardiovascular system is highly vulnerable to age-related functional decline, where the epigenetic-RCD axis emerges as a central orchestrator of pathological remodeling and functional deterioration. Epigenetic reprogramming serves as a critical interface that translates the cumulative burden of pathogenic stressors into a persistent recalibration of cell death pathways. This reprogramming drives the progression of diverse cardiovascular pathologies by coordinately disrupting core components of RCD.

The dysregulation of autophagy is a hallmark of CVDs, finely tuned by epigenetic cues. DNA methylation and histone modifications serve as important regulatory mechanisms. In AS, *GALECTIN-8* DNA methylation enhances macrophage autophagy via the MAPK/mTOR pathway to ameliorate the disease [173]. In addition, *lncARF* is activated through DNA hypomethylation at its promoter. This hypomethylation is mediated by FosB, which inhibits DNMT1 from binding to the *lncARF* promoter region. Once activated, lncARF suppresses foam cell autophagy by operating through the RRAGD/PI3K/AKT and MAPK pathways [174]. In diabetic AS, Sirt6-mediated Caveolin-1 acetylation activates the autophagic degradation pathway, inhibiting LDL transcellular transport [175]. In heart failure, DNMT1 enhances methylation of the *miR-152-3p* promoter to inhibit its expression, thereby inhibiting mitochondrial autophagy via the ETS1/RHOH signaling axis, ultimately aggravating the condition [176]. The histone demethylase KDM4A suppresses fibroblast autophagy, induces their premature senescence, and ultimately triggers post-MI interstitial fibrosis by regulating H3K9me3 modification in the *TRIM44* promoter region [177]. DNMT3A influences autophagy and the progression of cardiac fibrosis by suppressing *miR-200b* expression [178].

DNA methylation and histone modifications are involved in governing ferroptosis in cardiovascular diseases. In myocardial ischemia/reperfusion injury, DNMT1 can downregulate *MGST1* expression by mediating methylation of the *MGST1* promoter, thereby promoting ferroptosis [179]. Furthermore, KMT2B exacerbates ferroptosis in myocardial ischemia/reperfusion injury by upregulating H3K4 methylation levels to promote *RFK* gene transcription [180].

In myocardial fibrosis, DNA methylation exerts a notable regulatory effect on pyroptosis. Aberrant upregulation of *DNMT3A* and promoter hyper-methylation-mediated suppression of *MTHFR* expression are key epigenetic features; knocking down *DNMT3A* ameliorates pyroptosis and fibrosis [181]. The DNMT-lncRNA-NLRP3 regulatory axis constitutes a crucial program: DNMT3A reduces lncRNA *NEAT1* expression via methylation, while DNMT1 silences lncRNA *GAS5*, both of which facilitate NLRP3 pathway activation, thereby driving cardiac fibroblast pyroptosis and cardiac fibrosis [182, 183].

Epigenetic modifications govern necroptosis, thereby regulating the progression of cardiovascular pathology. In AS, the circadian rhythm-associated lncRNA AK023617 regulates the diurnal rhythm of necroptosis to establish a "macrophage death clock," leading to plaque instability [184]. Studies in a mouse model of MI demonstrate that circCacna1c alleviates myocardial necroptosis through inhibiting the HNRNPF/RIPK1 pathway [185], and CircRNA CNEACR inhibits necroptosis via the HDAC7/FOXA2/RIPK3 axis [186]. Cuproptosis, a newly discovered pathway, is implicated in myocardial injury under epigenetic control. Elevated Cu ion levels post-MI specifically trigger cardiomyocyte cuproptosis. Upregulated LRP6 promotes Cu ion influx and, through ALKBH5, inhibits the m6A modification of *FDX1*, thereby stabilizing *FDX1* expression and exacerbating cell death [187].

Collectively, these findings establish the epigenetic-RCD axis as a pervasive regulatory network that drives cardiovascular pathogenesis. The interplay among epigenetically controlled cell death pathways suggests that therapeutic success may require a paradigm shift from single-pathway inhibition to multi-pronged strategies that recalibrate the entire network, thereby achieving comprehensive cardioprotection.

### 4.5 Epigenetic modification of RCD in neurodegenerative diseases

Neurodegenerative diseases, including Alzheimer's disease (AD) and Parkinson's disease (PD), are driven by the progressive dysfunction and loss of specific neuronal populations, where multiple epigenetically regulated RCD pathways act as collective drivers of the disease process.

The dysregulation of autophagy is extensively remodeled by the epigenetic landscape in neurodegenerative diseases. In AD, cellular and animal model studies indicate that the histone demethylase KDM1A upregulates *METTL3* expression, thereby stabilizing the mRNA of the E3 ubiquitin ligase *STUB1* through the m6A-IGF2BP1-mediated pathway. This enhances the autophagic clearance of phosphorylated Tau protein (p-Tau) and ultimately delays AD pathological progression [188]. In addition, downregulated lncRNA *EPB41L4A-AS1* suppresses the activity of the histone acetyltransferase GCN5L2, reducing histone acetylation, crotonylation, and lactylation modifications in the promoter regions of autophagy-related genes, which inhibits their transcription and ultimately impedes Aβ clearance in glial cells [189]. In PD, multiple animal models suggest that Aconitase 2 (*ACO2*) insufficiency heightens susceptibility to the disease by disrupting histone acetylation to suppress autophagy gene transcription [190]. Notably, the tRNA-derived fragment tRF-02514 in extracellular vesicles inhibits autophagy by targeting *ATG5* in PD, whereas inhibiting tRF-02514 restores autophagy and delays disease progression [191].

Concurrently, epigenetically orchestrated ferroptosis emerges as a potent driver of neuronal damage, particularly in PD. Soot nanoparticles (SNPs) facilitate ferroptosis in dopaminergic neurons through modulating RNA m6A methylation. Specifically, SNPs elevate *ACSL4* expression by augmenting YTHDF1 - driven m6A modification within the 5'UTR of the *ACSL4* gene. This process expedites ferroptosis in dopaminergic neurons and contributes to the advancement of PD [192].

Cuproptosis is implicated in neuronal injury in AD under precise epigenetic control. In neuronal cells, PTBP1 significantly enhances cuproptosis and accelerates AD progression by upregulating *SLC31A1* expression and inhibiting its ubiquitination modification. The stability of PTBP1 experiences a further augmentation via m6A modification that is mediated by METTL3. In mouse models, inhibiting PTBP1 effectively prevents the occurrence of AD and alleviates ROS-mediated oxidative stress damage [193].

This collective evidence positions the epigenetic-RCD axis as a pathological amplifier and a unifying driver in neurodegenerative diseases. The axis drives a self-reinforcing cycle of cumulative cellular damage and neuronal loss through the coordinated dysregulation of RCD. Interrupting this cycle by targeting the core epigenetic machinery of the axis presents a unified strategy for neuroprotection.

### 4.6 Epigenetic modification of RCD in sarcopenia

The core pathology of sarcopenia involves muscle fiber degeneration driven by impaired ferroptosis inhibition and dysregulated autophagy. During the development of sarcopenia, downregulation of *GPRC5D-AS1* leads to reduced translational levels of SLC7A11 protein, whereas overexpression of *GPRC5D-AS1* upregulates *SLC7A11* to inhibit ferroptosis in human skeletal muscle cells, increasing their antioxidant capacity and viability [194]. DNA methylation within skeletal muscle exhibits an upward trend as aging progresses. Heightened DNA methylation levels in skeletal muscle perturb muscle homeostasis, resulting in reduced basal autophagy, exacerbation of skeletal muscle inflammation and aging traits, transformation of muscle fiber types toward slow-twitch fibers, and fast-twitch fiber-specific muscle atrophy [195]. Sarcopenia exemplifies systemic epigenetic deterioration that coordinately impairs protective autophagy and promotes susceptibility to ferroptosis. This suggests that countering age-related muscle decline requires broadly rejuvenating the epigenetic landscape to restore cellular homeostasis, rather than correcting individual pathways.

### 4.7 Epigenetic modification of RCD in cancer

Epigenetic reprogramming influences tumor progression, drug resistance, and cell death susceptibility by regulating multiple RCD pathways. Ferroptosis susceptibility is precisely modulated by epigenetic mechanisms. EZH2 exerts epigenetic suppression on *ATOH8* expression through facilitating DNA methylation within the *ATOH8* promoter region and elevating H3K27me3 levels, thereby inhibiting ATOH8-mediated negative regulation of stearoyl-CoA desaturase and ultimately reducing tumor cell susceptibility to ferroptosis [196].

Pyroptosis activation may also regulate tumor fate. *ZDHHC1*, downregulated by DNA methylation, significantly suppresses tumor growth by triggering oxidative/endoplasmic reticulum stress-mediated apoptosis and pyroptosis [197]. Cuproptosis is driven through the PVT1/*FDX1* axis. Research in colorectal cancer cells demonstrates that lncRNA PVT1 exhibits direct binding to the *FDX1* promoter, enhances H3K27ac deposition, and triggers *FDX1* transcription, thereby facilitating cuproptosis in colorectal cancer [198].

Epigenetically regulated necroptosis influences tumor progression. The buildup of myeloid - derived suppressor cells (MDSCs) serve as a characteristic feature of cancer. MDSCs construct an autocrine IL6-STAT3-DNMT epigenetic pathway to repress *TNFα* expression. Consequently, they inhibit TNFα-triggered RIP1-dependent necroptosis and sustain cellular viability and aggregation [199]. lncRNA HABON suppresses hypoxia - triggered necroptosis in hepatocellular carcinoma cells through interacting with VDAC1 and modulating the opening state of the mitochondrial permeability transition pore (mPTP) [200].

Epigenetic modification-autophagy axis influences tumor progression and chemotherapy response. Nicotinamide nucleotide transhydrogenase (NNT) enhances cisplatin sensitivity in lung cancer by suppressing protective autophagy. Moreover, hypermethylation of the *NNT* DNA is directly associated with an unfavorable prognosis in patients with NSCLC. Targeted demethylation of *NNT* substantially lowered its DNA methylation status, impeded cellular autophagy, and diminished cisplatin resistance [201].

In cancer, the epigenetic-RCD axis is a central determinant of cell fate, capable of being manipulated by the tumor for survival or leveraged by therapy for eradication. This plasticity dictates that the future of oncology lies in strategically steering the entire axis. A dual approach should be adopted to overcome drug resistance by suppressing its pro-tumorigenic outputs while simultaneously activating its onco-suppressive potentials.

## 5. Targeting RCD pathways through epigenetic modulation for aging-related diseases

### 5.1 Non-pharmacological treatment

Lifestyle interventions can delay aging-related diseases by modulating the epigenetic- regulated cell death (RCD) axis (Fig. 4). A growing body of evidence indicates that exercise, serving as a potent epigenetic regulator, can extensively recalibrate the network of RCD and improve aging-related diseases. Exercise inhibits endothelial cell pyroptosis and alleviates AS by downregulating METTL14-mediated N6-methyladenosine (m6A) methylation of lncRNA NEAT1 [202]; additionally, exercise enhances autophagy and improves heart failure by suppressing lncRNA MALAT1 to induce the activation of the PI3K/AKT pathway [203]. Research has demonstrated that exercise alleviates diabetic cardiomyopathy by inhibiting ferroptosis mediated by N-Acetyltransferase 10 [204]. The activation of NRF2 and BDNF induced by physical exercise also represents a potentially effective strategy for regulating ferroptosis in Parkinson's disease (PD) [205]. Exercise has the ability to prompt changes in DNA methylation across the entire genome, particularly impacting genes involved in energy control, mitochondrial activities, biosynthetic processes, as well as those associated with muscle repair, calcium signal transmission, and brain adaptability [206, 207]. Exercise promotes the shift of cell fate towards survival and homeostasis by inducing epigenetic changes, thereby enhancing the resistance of various tissues to multiple forms of RCD.

In nutritional interventions, a mixture of algae and extra virgin olive oils attenuates aging-induced decline in autophagic activity and downregulates factors associated with muscular aging—including histone deacetylase (HDAC) 4, myogenin, and IGFBP-5—while effectively preventing aging-triggered declines in the mass of gastrocnemius muscle, overall protein quantity, and the expression levels of myosin heavy chain mRNA [208]. In addition, folic acid supplementation may prevent and treat aging-related diseases through the epigenetics-RCD axis. Hypermethylation of the *MTHFR* promoter region leading to reduced expression and consequent cellular pyroptosis represents an important epigenetic feature of diabetic myocardial fibrosis. Folic acid supplementation can reverse this process and prevent diabetic cardiac fibrosis [181]. Furthermore, folic acid downregulates *ALKBH5* expression by inducing hypermethylation of its promoter, leading to increased m6A modification and upregulated expression of *ATG12*, thereby promoting autophagy and counteracting hepatic steatosis [209].

**Figure 4.**
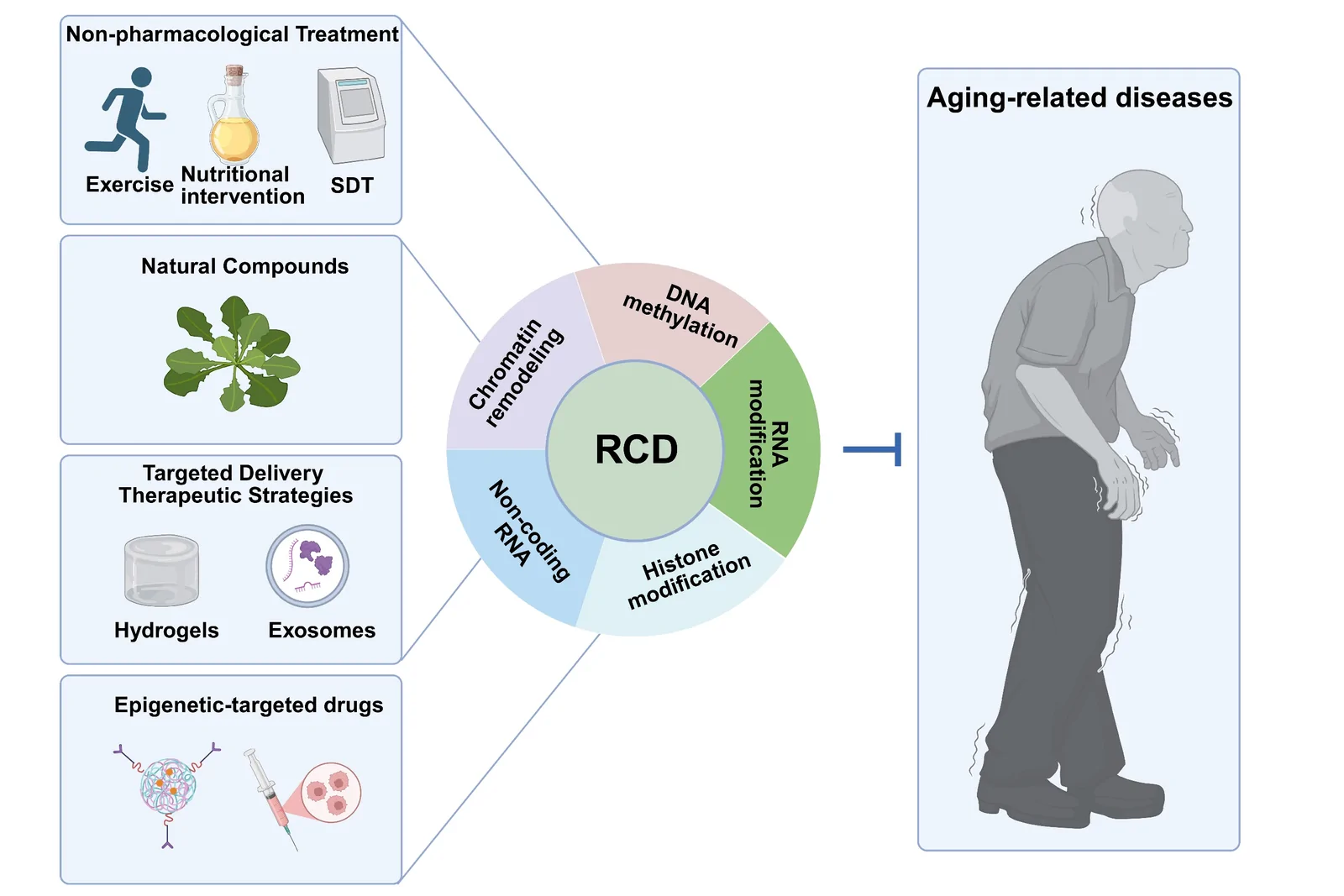
**Therapeutic targeting of the epigenetics-RCD axis in aging-related diseases**. Therapeutic strategies targeting the epigenetic-RCD axis—including non-pharmacological treatments, natural compounds, targeted delivery therapeutic strategies, and epigenetic-targeted drugs—play a role in postponing the emergence of aging-related diseases. The efficacy of these interventions has been demonstrated in preclinical models of aging-related cardiovascular, metabolic, and neurodegenerative diseases, evaluated through molecular, functional, and behavioral analyses.

Sonodynamic therapy (SDT), an emerging medical technology, achieves therapeutic effects through localized activation of preloaded sonosensitizers by low-intensity ultrasound. Research findings demonstrate that SDT mitigates myocardial apoptosis triggered by myocardial infarction (MI) through the activation of lncRNA MHRT-mediated autophagy, indicating its potential as a therapeutic strategy for MI [210]. SDT confers cardioprotection via the epigenetic-autophagy axis, a mechanism similarly observed in ischemic postconditioning (IPostC). IPostC upregulates *miR-30a* expression by regulating DNA methylation levels at the *miR-30a* promoter region through DNMT3b, thereby suppressing BECN1-dependent autophagy and protecting aged cardiomyocytes from hypoxia/reoxygenation injury [211]. These non-pharmacological strategies coordinate RCD balance through epigenetic reprogramming, providing foundational support for aging intervention. These findings collectively demonstrate that non-pharmacological interventions can achieve precise, physiological reprogramming of the epigenetic-RCD axis, providing foundational support for aging intervention. Non-pharmacological strategies serve as a cornerstone that enhances efficacy and reduces the burden of future pharmaceutical interventions.

### 5.2 Natural compounds and derived drugs

Natural compounds, through precisely targeting key epigenetic-modifying factors, dynamically regulate RCD pathways, thereby presenting a groundbreaking therapeutic avenue for addressing aging - associated diseases (Fig. 4 and Fig. 5).

In metabolic diseases, arbutin alleviates the pathological progression of NAFLD by suppressing the activity of the RNA demethylase FTO, thereby enhancing m6A methylation of *SLC7A11* mRNA and inhibiting the ferroptosis pathway [212]. Furthermore, chlorogenic acid (CGA) binds to and inhibits the m6A demethylase activity of ALKBH5, restoring autophagic homeostasis to improve hepatic lipid deposition [213]. (-)-epigallocatechin 3-gallate (EGCG) can degrade FTO, increase m6A modification on the mRNA of pro-oxidative genes (*TLR4*, *RELA*, *SRC*), reduce ROS accumulation, protect pancreatic β-cells from damage induced by excessive autophagy, and delay the progression of diabetes [214]. In DKD, rutin inhibits HDAC1 through the PI3K/AKT/mTOR signaling cascade, thereby reinstating autophagic processes to mitigate endothelial-mesenchymal transition [215]. Cranberry extract improves diabetic cognitive dysfunction by downregulating *GAS5* and inhibiting the pyroptosis pathway [216]. Lentinus edodes mycelia polysaccharide (LMP) antagonizes advanced glycation end products-induced endothelial cell pyroptosis and improves diabetic vascular complications through modulation of the MALAT1/miR-199b/mTOR axis along with the NLRP3/Caspase-1/GSDMD cascade [217].

**Figure 5.**
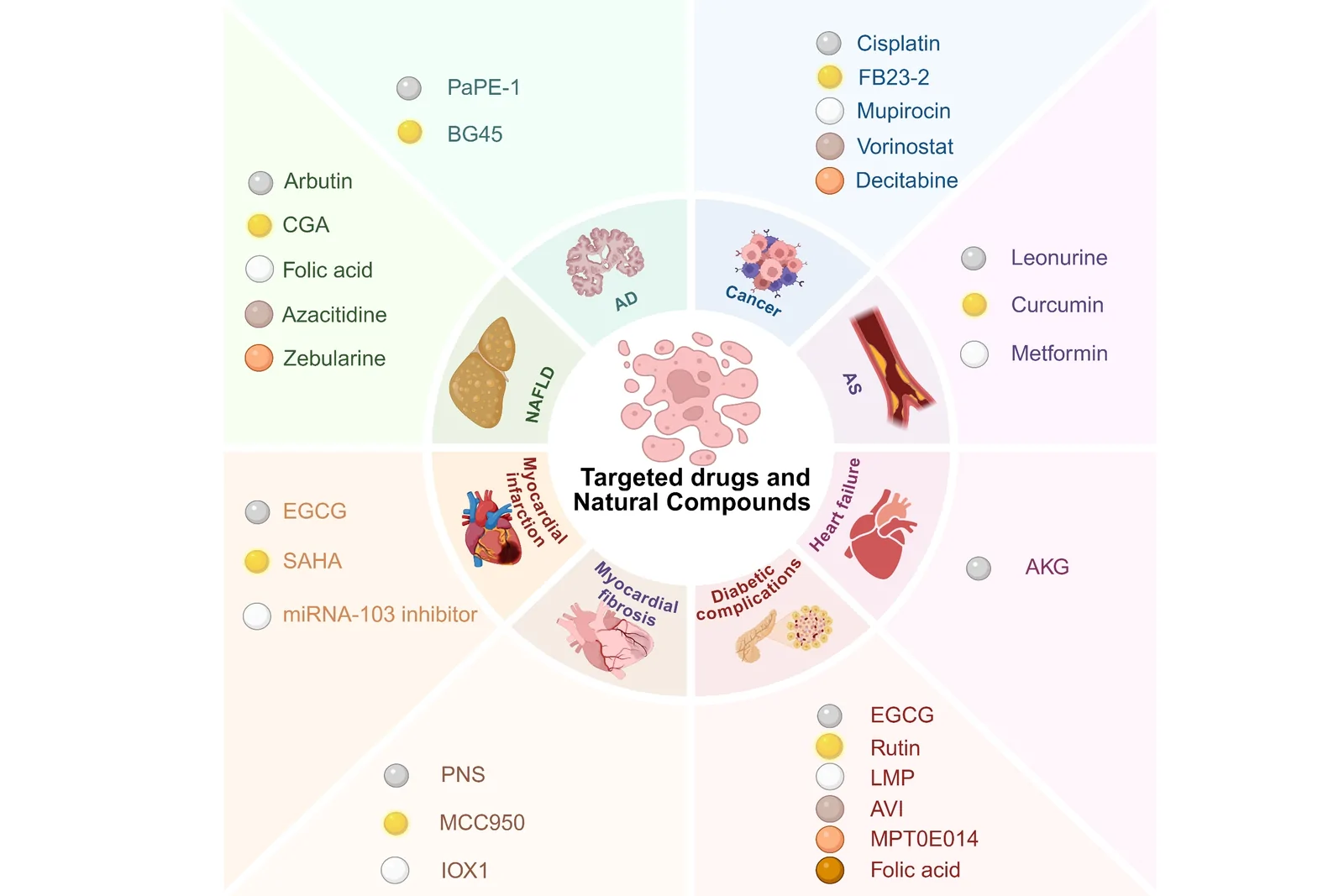
**Therapeutic drugs targeting the epigenetic-RCD axis against different aging-related diseases**. Multiple therapeutic agents targeting the epigenetic-RCD axis demonstrate efficacy in preventing and treating aging-related pathologies, including AD, NAFLD, myocardial infarction, myocardial fibrosis, diabetic complications, heart failure, AS, and cancer. Specific agents and their corresponding disease targets are illustrated in the diagram. The therapeutic effects are supported by data from respective cellular and animal disease models, as well as, for some agents like Metformin and Cisplatin, evidence from clinical studies.

In the field of cardiovascular diseases, the multidimensional modulatory impacts of natural products are particularly significant. In AS models, leonurine improves AS by regulating METTL3-mediated *AKT1S1* mRNA stability, activating foam cell autophagy, and promoting metabolic reprogramming [218]; curcumin significantly enhances autophagic activity in podocytes by inhibiting mTOR to promote TFEB nuclear translocation, reduces ROS levels, inhibits P300 activity, thereby decreasing histone acetylation levels, and ultimately alleviates inflammatory responses during AS formation [219]. Furthermore, supplementation with α-Ketoglutarate (AKG) improves heart failure by activating the SIRT-PINK1 and SIRT1-GPX4 signaling axes, facilitating mitophagy and suppressing myocardial ferroptosis [220]. Panax notoginseng saponins (PNS) ameliorate cardiac function and attenuate myocardial fibrosis in cardiorenal syndrome type 4 rats by suppressing cardiomyocyte pyroptosis via down-regulation of lncRNA *ANRIL* [221]. Notably, beyond delaying diabetes progression, EGCG also inhibits myocardial pyroptosis via MEG3/TAF15/AIM2 axis, suggesting its potential as an innovative therapeutic candidate for MI and highlighting its multi-system protective effects against aging-related diseases [222].

The multifaceted actions of natural compounds validate the strategy of multi-target engagement against the epigenetic-RCD axis. They serve as pioneering therapeutic leads that provide a rational basis for developing novel, mechanism-based multi-targeted drugs to combat aging-related diseases.

### 5.3 Targeted delivery therapeutic strategies

Carrier-engineered strategies mediating targeted interventions in the epigenetic-RCD network provide transformative treatment paradigms for multi-organ aging-related diseases (Fig. 4). The LRP6 protein exacerbates cuproptosis by promoting Cu ion influx and suppressing m6A modification of *FDX1*. The novel LRP6 inhibitor chrysin-7-O-glucuronide (C7Og) achieves targeted delivery via Janus hydrogels. In rat and Bama minipig models of MI, the delivery system markedly facilitates myocardial repair while enhancing cardiac performance and electrical signal transmission, thereby proposing an innovative strategy with potential clinical value for MI treatment [187]. CAY10603 is a potent and selective HDAC6 inhibitor. The brain-targeted nanocarrier loaded with this HDAC6 inhibitor exerts neuroprotective effects by reversing mitochondrial dysfunction, suppressing reactive oxygen species generation, and inhibiting α-synuclein accumulation [223]. Simultaneously, stem cell-derived exosomes coordinate multi-organ RCD balance through epigenetic reprogramming: mesenchymal stem cell-derived HIF-1α-overexpressed extracellular vesicles activate YTHDF1-mediated protective autophagy, effectively mitigating hypoxia-triggered apoptosis and cellular senescence in pancreatic β-cells, thereby representing a promising novel therapeutic strategy against hypoxic cytotoxicity in pancreatic β-cells [224]. The BMSC-derived exosome loaded with lncRNA GAS5 inhibits ferroptosis by regulating the ULK1/Hippo pathway, thereby exerting therapeutic effects against heart failure [147]. Furthermore, the lncRNA XIST, which is carried by extracellular vesicles derived from adipose tissue-derived mesenchymal stem cells, inhibits cardiomyocyte pyroptosis in atrial fibrillation by preventing the miR-214-3p-mediated suppression of *ARL2* [163]. The success of targeted delivery systems proves that the principal challenge of therapeutic specificity is solvable. By enabling spatiotemporal precision, these strategies transform the epigenetic-RCD axis from a compelling theoretical target into a clinically tractable reality, setting a new standard for future drug development in aging-related diseases.

### 5.4 Epigenetic-targeted drugs

Epigenetic-targeted drugs remodel RCD balance by precisely regulating key modifying enzymes (e.g., methyltransferases, demethylases, histone-modifying enzymes) and their downstream pathways, thereby providing mechanism-oriented therapeutic strategies for aging-related diseases (Fig. 4 and Fig. 5).

In the fields of cardiovascular and neurodegenerative diseases, targeting the epigenetic-RCD axis demonstrates significant potential. The HDAC inhibitor suberoylanilide hydroxamic acid (SAHA) alleviates ischemia/reperfusion injury, mitigates MI and improves cardiac function by inducing cardiomyocyte autophagy [225]. The hypoglycemic drug metformin inhibits AS progression by regulating lncRNA *TUG1* to activate the AMPK-mTOR pathway and induce protective autophagy [226]. The *miRNA-103* inhibitor antagonizes necroptosis and mitigates MI injury by targeting the FADD/RIPK pathway [227]. Small molecule inhibitors targeting the ALKBH5-NLRP3 loop, such as MCC950 or IOX1, alleviate cardiomyocyte pyroptosis, improve cardiac dysfunction, and reduce myocardial fibrosis [118]. Furthermore, the non-nuclear estrogen receptor signaling agonist pathway preferential estrogen-1 (PaPE-1) exerts neuroprotective effects by reversing Aβ-induced autophagy inhibition, stimulating the expression of autophagy-related genes (*BECN1*, *ATG5*, *AMBRA1*, *MAP1LC3B*), and reducing DNA methylation levels of genes including *ATG7* [228]. In addition, the Class I HDAC inhibitor BG45 can reduce Aβ deposition and rescue synaptic damage and neuron loss [229]. Thus, PaPE-1 and BG45 may represent a novel therapeutic approach for AD.

Epigenetic-targeted drugs demonstrate significant efficacy in preventing and treating diabetic complications as well as NAFLD. The HDAC inhibitor MPT0E014 improves cardiac function and delays the progression of DCM in T2DM animal models by modulating autophagy, inflammation, and insulin signaling pathways [230]. In DOP, Asperosaponin VI (AVI) antagonizes ferroptosis and restores bone homeostasis by inhibiting DNMT1/3a-mediated hypermethylation of the *GPX4* promoter, alleviating DOP [171]. The DNMT1 inhibitors azacitidine and zebularine restored autophagy and the M1/M2 polarization balance in Kupffer cells, thereby preventing the progression of NAFLD [231].

In cancer therapy, epigenetic-targeted drugs enhance therapeutic efficacy by regulating RCD. Cisplatin exerts anticancer effects by upregulating lncRNA *MEG3* to activate the NLRP3/caspase-1/GSDMD axis, inducing pyroptosis in triple-negative breast cancer cells [166]; FTO impedes autophagy and promotes the progression of clear cell renal cell carcinoma (ccRCC) through an m6A-IGF2BP2-dependent pathway. The FTO-targeted small-molecule inhibitor FB23-2 markedly suppresses tumor growth and extends survival in patient-derived xenograft (PDX) models. These findings imply that FTO inhibitors hold promise as effective therapeutic agents for ccRCC [232]. Furthermore, the novel FTO inhibitor mupirocin induces ferroptosis in colorectal cancer cells and inhibits tumor growth, while significantly enhancing the antitumor effects of ferroptosis inducers erastin or RSL3. This indicates that targeted inhibition of FTO by mupirocin not only represents a potentially effective therapeutic modality for colorectal cancer but also potentiates the chemotherapeutic efficacy of erastin and RSL3 [109]. The HDAC inhibitor vorinostat can inhibit the deacetylation of key autophagic markers, thereby promoting autophagic cell death and leading to the demise of cancer cells [223]. In addition, HDAC inhibitors can activate lipid peroxidation and ferroptosis in gastric cancer [233]. The DNMT inhibitor decitabine can induce apoptosis and activate autophagy in myeloid leukemia by downregulating *TIGAR* [234].

Epigenetic-targeted drugs represent a mechanism-based therapeutic paradigm. By precisely regulating key modifying enzymes and their mediated RCD programs, they remodel cellular fate balance, directly address the pathological cores of aging-related diseases, and thereby paving the way for next-generation root-targeted precision therapies.

### 5.5 Status of clinical translation

The translational feasibility of the fundamental scientific concept of the epigenetic-RCD axis into clinical practice has gained robust support from clinical medicine. Numerous drugs targeting the epigenetic-RCD axis have well-established use in treating aging-related diseases. For instance, extensive clinical studies have established that metformin, which can induce protective autophagy by modulating lncRNAs, improves cardiovascular outcomes in addition to its glucose-lowering effects [235-238]. The chemotherapeutic agent cisplatin, which can induce pyroptosis through epigenetic regulation, is widely used in cancer treatment and extends patient survival [239, 240]. In addition, approved drugs that directly target the epigenetic machinery, such as HDAC inhibitors (vorinostat, panobinostat) and DNMT inhibitors (decitabine, azacitidine), directly demonstrate through their antitumor activity that targeting epigenetics can alter cell fate and improve patient outcomes in a clinical setting [241-244].

This body of evidence anchors the therapeutic potential of the epigenetic-RCD axis for aging-related diseases from a theoretical concept into clinical reality, providing an evidence-based foundation for optimizing existing therapies and expanding new indications based on this axis. Beyond therapeutic strategies, exploiting the dynamic changes of this axis to develop novel biomarkers is regarded as a crucial translational pathway toward precision medicine. Circulating epigenetic regulators and RCD-specific signals demonstrate considerable potential as tools for non-invasive diagnosis, prognostic assessment, and treatment response prediction. However, clinical research that directly and systematically targets this specific axis remains relatively scarce, representing a critical direction for future research efforts.

## 6. Conclusion and future perspectives

The intricate crosstalk between epigenetic modifications and regulated cell death (RCD) pathways constitutes an important axis driving aging and aging-related diseases. Aging-associated remodeling of the epigenetic landscape profoundly influences the activation or inhibition thresholds of RCD pathways, disrupts cellular homeostasis, and drives tissue functional decline and pathological progression. Targeting specific epigenetic factors (e.g., methylation/demethylation enzymes, histone-modifying enzymes, non-coding RNAs) has proven capable of precisely modulating RCD pathways, revealing substantial potential for intervening in aging and aging-related diseases. These findings underscore the central role of epigenetic modification-regulating molecules as "molecular switches" that reprogram RCD pathways to influence tissue homeostasis, thereby laying the theoretical groundwork for intervening in aging processes.

Despite the transformative potential of targeting the epigenetic-RCD axis for intervening in aging and related diseases, the path from concept to clinical translation remains fraught with systemic challenges that require urgent resolution. The primary challenges stem from the inherent complexity of epigenetic regulation and current technological limitations. The dual nature of this axis—whereby the same epigenetic modification can exert diametrically opposite effects on specific RCD pathways (such as autophagy or pyroptosis) across different tissues or disease stages—demands intervention strategies with extremely high tissue specificity and stage restriction. However, achieving such precision is exceptionally challenging due to significant off-target risks; both CRISPR/dCas9-based epigenetic editors and small-molecule inhibitors may interact with non-target genomic sites, leading to unpredictable gene expression dysregulation and unforeseen toxic side effects in complex organisms. Secondly, technological bottlenecks in drug delivery systems pose another critical barrier. Efficient in vivo delivery is crucial for therapeutic success, yet current technologies for specifically, efficiently, and safely delivering macromolecular editors, nucleic acid drugs, or small-molecule compounds to target organs and specific cell subtypes remain underdeveloped, severely limiting therapeutic efficacy. Furthermore, interindividual epigenetic heterogeneity constitutes a fundamental challenge in translational medicine. The unique epigenetic landscape of each individual, shaped by genetic background, age, and lifelong environmental exposures (e.g., nutrition, toxins), means that uniform therapies may elicit vastly divergent responses—from highly effective to ineffective or even harmful—creating significant difficulties for patient recruitment in clinical trials, efficacy evaluation, and the development of universal treatment protocols.

To overcome these hurdles, a multi-pronged strategy is essential. Technologically, developing high-fidelity epigenetic editing tools and intelligent targeted delivery systems is central to enhancing safety and efficacy. Strategically, harnessing biomarkers based on this axis is regarded as a critical pathway to achieving precision medicine and breaking through translational bottlenecks. Such biomarkers hold immense potential as powerful tools for non-invasive early diagnosis, dynamic therapeutic monitoring, and precise patient stratification. However, their development itself faces challenges in standardization, validation, and biological specificity. For instance, how to distinguish disease-specific RCD signals from physiological cell turnover, and how to validate their predictive value in large prospective cohorts. Nevertheless, by deeply integrating such biomarkers with multi-omics data (epigenomic, transcriptomic, proteomic) and leveraging advanced computational models and artificial intelligence to construct individualized network maps, we can identify the most core and actionable regulatory hubs. Ultimately, through rigorous, stepwise clinical validation that carefully considers long-term safety, off-target effects, and individual heterogeneity, we can successfully transform RCD from a passive disease endpoint into a precisely regulable therapeutic node, thereby truly inaugurating a new era for aging intervention and chronic disease management.

The mechanistic framework of the epigenetic-RCD axis remains constrained by its reliance on experimental approaches with inherent limitations. The heavy reliance on high-throughput methodologies (e.g., ChIP-seq, MeRIP-seq) and association studies (e.g., RIP-qPCR) presents challenges, including antibody specificity in immunoprecipitation, batch effects in sequencing, and the inherent risk of false positives. Many mechanistic insights are derived from gain/loss-of-function approaches in simplified cellular or induced aging models (e.g., progeroid mice, toxin-induced disease models), which may not fully capture the chronic, multi-factorial nature of human aging. A significant translational gap exists, as direct evidence validating these specific epigenetic-RCD interactions in aged human tissues or longitudinal human cohorts is still largely lacking. For instance, the pathophysiological roles of key axes discussed in this Perspective-such as the DNMT1/MGST1/ferroptosis axis in the heart following ischemia-reperfusion injury, KDM1A/METTL3/autophagy axis in Alzheimer's disease, and the SNHG14/miR-493-5p/MEF2C/autophagy axis in osteoporotic bone-await definitive confirmation in relevant human tissues and clinical studies. Moving forward, bolstering the robustness and clinical relevance of this axis requires a concerted shift in two key directions: First, towards causal validation using precise epigenetic editing tools in more physiologically relevant disease models. Second, it is paramount to pursue independent corroboration in human samples, leveraging biobanks, multi-omics data from aging cohorts, and ideally, interventional clinical studies. Addressing these methodological and translational considerations will be paramount for transforming the epigenetic-RCD paradigm from a compelling theoretical construct into a source of reliable diagnostics and therapeutics for human aging and its related diseases.

Aging is not merely linear functional decline, but the outcome of intricate interplay between epigenetic programs and cell death pathways. Future research should transcend molecular-level dissection to construct a dynamic "epigenetic-RCD-aging" interaction network model, ultimately achieving seamless transition from mechanistic elucidation to clinical translation. The intersection of epigenetics and RCD emerges as a pivotal bridge connecting fundamental research with translational medicine, offering new hope for postponing the aging process and averting/addressing aging-related diseases.

## Glossary of key terms

DNA Methylation: DNA methylation primarily modifies cytosines within CpG dinucleotides, resulting in 5-methylcytosine.
Histone Modifications: Key types of histone modifications are methylation, acetylation, phosphorylation, ubiquitination, and ADP-ribosylation, among others.
Chromatin Remodeling: Chromatin remodeling refers to the dynamic alteration of chromatin architecture and spatial organization across the genome.
Non-coding RNAs: Functional RNA molecules that are not translated into proteins, which regulate gene expression at transcriptional and post-transcriptional levels.
RNA Methylation: Chemical modifications to RNA molecules, with N6-methyladenosine (m6A) being the most prevalent, which regulate RNA splicing, stability, transport, and translation.
Pyroptosis: Pyroptosis is a programmed cell death process initiated by inflammasomes and mediated by gasdermin proteins, which ultimately leads to cell lysis through pore formation in the plasma membrane.
Ferroptosis: Ferroptosis represents a distinctive form of RCD that is iron-dependent and features the toxic buildup of lipid peroxides, resulting in oxidative destruction of cellular membranes.
Cuproptosis: Cuproptosis is a modality of cell death characterized by excessive intracellular Cu accumulation. This process is triggered when Cu induces the aggregation of mitochondrial enzyme dihydrolipoamide S-acetyltransferase, disrupting the TCA cycle and Fe-S cluster proteins, ultimately leading to proteotoxic stress and cell death.
Autophagy: Autophagy, a cellular degradation process essential for maintaining homeostasis, is primarily categorized into three distinct forms: microautophagy, macroautophagy, and chaperone-mediated autophagy.
Necroptosis: Necroptosis represents an RCD pathway mediated by the kinase activity of receptor-interacting serine/threonine-protein kinase 1 (RIPK1), which orchestrates the assembly of complex IIB to execute cell necroptosis.
